# Pharmacological Modulation by Low Molecular Weight Heparin of Purinergic Signaling in Cardiac Cells Prevents Arrhythmia and Lethality Induced by Myocardial Infarction

**DOI:** 10.3390/jcdd10030103

**Published:** 2023-02-27

**Authors:** Carlos Eduardo Braga Filho, Adriano Henrique Pereira Barbosa, Lucas Antonio Duarte Nicolau, Jand Venes Rolim Medeiros, Marcelo Pires-Oliveira, Rui Manuel dos Santos Póvoa, Tânia Carmen Penãranda Govato, Hézio Jadir Fernandes Júnior, Rafael Guzella de Carvalho, Bráulio Luna-Filho, Fernando Sabia Tallo, Erisvaldo Amarante de Araújo, José Gustavo Padrão Tavares, Ricardo Mario Arida, Afonso Caricati-Neto, Francisco Sandro Menezes-Rodrigues

**Affiliations:** 1 Postgraduate Program in Cardiology, Universidade Federal de São Paulo (UNIFESP), São Paulo 04024-000, SP, Brazil; 2 Department of Biotechnology, Universidade Federal do Delta do Parnaíba (UFDPar), Parnaíba 64202-020, PI, Brazil; 3 União Metropolitana de Educação e Cultura–School of Medicine (UNIME), Lauro de Freitas 42700-000, BA, Brazil; 4 Department of Urgency and Emergency Care, Universidade Federal de São Paulo (UNIFESP), São Paulo 04024-000, SP, Brazil; 5 Department of Pharmacology, Universidade Federal de São Paulo (UNIFESP), São Paulo 04023-062, SP, Brazil; 6 Department of Physiology, Universidade Federal de São Paulo (UNIFESP), São Paulo 04023-062, SP, Brazil

**Keywords:** low-molecular weight heparin, enoxaparin, cardiac ischemia/reperfusion, cardiac arrhythmias, purinergic signaling

## Abstract

Background: Although several studies suggest that heparins prevent arrhythmias caused by acute myocardial infarction (AMI), the molecular mechanisms involved remain unclear. To investigate the involvement of pharmacological modulation of adenosine (ADO) signaling in cardiac cells by a low-molecular weight heparin (enoxaparin; ENOX) used in AMI therapy, the effects of ENOX on the incidences of ventricular arrhythmias (VA), atrioventricular block (AVB), and lethality (LET) induced by cardiac ischemia and reperfusion (CIR) were evaluated, with or without ADO signaling blockers. Methods: To induce CIR, adult male Wistar rats were anesthetized and subjected to CIR. Electrocardiogram (ECG) analysis was used to evaluate CIR-induced VA, AVB, and LET incidence, after treatment with ENOX. ENOX effects were evaluated in the absence or presence of an ADO A1-receptor antagonist (DPCPX) and/or an inhibitor of ABC transporter-mediated cAMP efflux (probenecid, PROB). Results: VA incidence was similar between ENOX-treated (66%) and control rats (83%), but AVB (from 83% to 33%) and LET (from 75% to 25%) incidences were significantly lower in rats treated with ENOX. These cardioprotective effects were blocked by either PROB or DPCPX. Conclusion: These results indicate that ENOX was effective in preventing severe and lethal arrhythmias induced by CIR due to pharmacological modulation of ADO signaling in cardiac cells, suggesting that this cardioprotective strategy could be promising in AMI therapy.

## 1. Introduction

The World Health Organization (WHO) reports that about 17 million people annually die worldwide due to cardiovascular diseases (CVD), especially ischemic heart diseases (IHD) [1]. The duration of obstruction and extent of myocardial ischemia constitute the main determinants of AMI severity, as these factors can increase the incidence of fatal cardiac arrhythmias and myocardial tissue death and even lead to sudden death of the patient [2,3]. The use of coronary artery recanalization techniques, especially fibrinolytic drugs and percutaneous primary coronary intervention (PCIp), to reestablish coronary blood flow in patients with AMI, has considerably advanced the treatment of cardiac ischemia. Nevertheless, coronary reperfusion can also produce myocardial tissue damage as severe as that produced by ischemia, defined as myocardial reperfusion injuries [2,4]. Injuries caused by cardiac ischemia and reperfusion (CIR) can be fatal mainly due to increased incidences of cardiac arrhythmias, myocardial tissue death, and sudden death [2,3,4].

The use of animal models of CIR to reproduce AMI in the laboratory has contributed to the elucidation of molecular mechanisms and to the development of new cardioprotective strategies to prevent the death of patients affected by ischemic myocardial diseases. Reperfusion-induced cardiac injuries include arrhythmias, myocardial stunning, micro-vascular dysfunction, and cell death [3,5,6,7]. Oxidative stress, calcium overload, and inflammation have all been implicated as injury mechanisms, which act synergistically: oxidative stress can lead to calcium overload, and both can lead to cell death [5,6,7,8]. Phosphorylation of ryanodine receptor 2 (RyR2) by Ca^2+^ calmodulin-dependent kinase II (CaMKII) was previously shown as a key mechanism related to Ca^2+^ disfunction and arrhythmias in CIR [9], and, later, redox modifications of RyR2 were seen to concur with phosphorylation in arrhythmogenesis [10]. Phosphorylation of NaV1.5 by CaMKII may also increase pro-arrhythmogenic late Na+ currents [3,11,12,13]. Thus, pharmacological interventions presently used or investigated in reperfusion-induced injuries usually modulate Ca^2+^ signaling or have antioxidant and/or anti-inflammatory properties [3,5,14,15,16].

Heparins, including low-molecular weight heparins (LMWHs), in turn, reduce the incidence of CIR-induced arrhythmias, supporting the idea that this pharmacological cardioprotective strategy could be effective and safe in AMI therapy in humans [17,18,19]. However, the cellular and molecular mechanisms involved in this cardioprotective activity of heparins have not been completely understood.

LMWHs, including enoxaparin (ENOX), are largely used in AMI therapy due to their anticoagulant activity. In addition, LMWHs have other pharmacological actions including anti-inflammatory, antioxidant, anti-apoptotic, and cardioprotective actions [17,18,19]. Some studies also suggest that LMWHs act on plasmalemmal Ca^2+^ and Na^+^ transport in cardiac cells, reducing cellular excitability and preventing arrhythmias [17,18]. In our recent studies, we observed that LMWHs modulate cell signaling, regulating the intracellular concentration of second messengers in cardiac cells, especially Ca^2+^ ions, cyclic adenosine monophosphate (cAMP), and ADO, finely regulating the membrane excitability and contractility of these cells and thus the potentially arrhythmogenic effects of AMI [17]. In addition, our studies have demonstrated that LMWHs stimulate the extracellular transport of cAMP, which, in turn, is rapidly converted to ADO and stimulates A1-adenosine receptors (A1R) in cardiac cells, attenuating the positive chronotropic response [20,21], and, consequently, reducing the incidence of cardiac arrhythmias [17,22,23]. A1R are involved in cardioprotective responses in different physiological and pathological conditions, including CIR and cardiac pre- and post-ischemic conditioning [17,22,24]. To advance our understanding of the cardioprotective actions of LMWHs, we investigated whether treatment with ENOX is effective and safe to reduce the incidence of severe and fatal arrhythmias induced by CIR. We also analyzed whether pharmacological modulation of ADO signaling in cardiac cells is involved in these cardioprotective effects of ENOX.

## 2. Materials and Methods

### 2.1. Animals

This study was performed using male adult Wistar rats (12- to 14-weeks old) weighing 320 to 350 g, obtained from the Center for the Development of Animal Models for Medicine and Biology (CEDEME)/UNIFESP. Animals were maintained under standard conditions of nutrition, hydration, temperature, light, and humidity. All experimental protocols used in the present work were approved by the Ethics Committee of the Federal University of São Paulo (CEUA-UNIFESP #1130/11 and #0065/12).

### 2.2. Induction of Cardiac Ischemia and Reperfusion (CIR)

**Figure 1:**
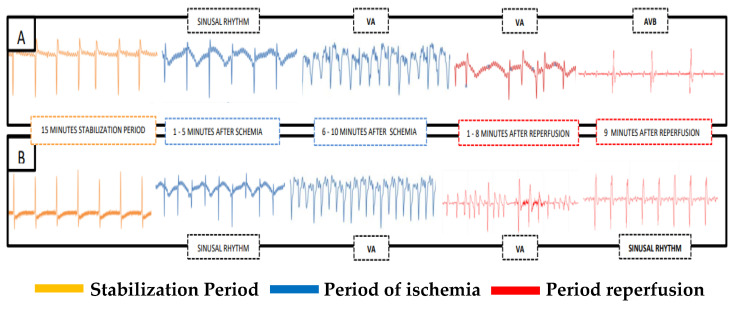
(**A**) ECG recording of animals submitted to the CIR without treatment with ENOX; (**B**) ECG recording of animals submitted to the CIR under treatment with ENOX, intravenously, immediately before performing cardiac ischemia due to occlusion of the left descending coronary artery. AV = Ventricular arrhythmia; AVB = Atrioventricular block; CIR = Cardiac ischemia and reperfusion.

The surgical procedures used for induction of CIR in rats were performed in accordance with methodology previously described by our research group [22,24]. Using this methodology, rats were anesthetized with urethane (1,25 g/kg, i.p.), intubated (Jelco 14G, Descarpack Descartáveis do Brazil LTDA, Ilhota, SC, Brazil), and mechanically ventilated (Insight model EFF 312 mechanical ventilator; Insight Equipamentos Científicos, Ribeirão Preto, SP, Brazil) [22,25,26]. After stabilization for 15 min, thoracotomy was performed to place a vascular tourniquet (5-0 braided silk suture attached to a 10-mm micropoint reverse cutting needle, (Mononylon Ethilon – Vascular; Atramat®, Coyoacán, México) around the left anterior descending coronary artery to induce ischemia. After 10 min of cardiac ischemia, the tourniquet was removed to allow coronary recirculation for 75 min (cardiac reperfusion) (Figure 1).

### 2.3. Evaluation of Cardiac Activity during CIR

To evaluate the effects of ENOX on cardiac electrical activity in rats subjected to CIR, all animals were ECG-monitored as previously described [25,26]. ECG analysis was performed for 100 min (stabilization for 15 min, cardiac ischemia for 10 min, and cardiac reperfusion for 75 min) to evaluate the incidence of ventricular arrhythmias (VA), atrioventricular block (AVB), and lethality (LET) caused by CIR (Figure 1). ECG was recorded using a biopotential amplifier by means of needle electrodes placed subcutaneously on the limbs. Successful surgical obstruction of the coronary artery was validated by ECG alterations (increase in R wave and ST segment) caused by CIR [14]. Body temperature was maintained at 37.5 °C with a heated operating platform and appropriate heating lamps and was routinely evaluated with a rectal thermometer [22,23]. ECG data were recorded with the AqDados 7.02 acquisition system (Lynx Tecnologia Ltda, São Paulo, SP, Brazil) and analyzed with the AqDAnalysis 7 software (Lynx Tecnologia Ltda, São Paulo, SP, Brazil). Using this software, we evaluated heart rates and incidence of VA, AVB, and LET resulting from CIR. Ventricular fibrillation, *torsades de pointes*, and ventricular tachycardia were all considered as VA [22,24]. 

### 2.4. Biochemical Evaluation of Biomarkers of Cardiac Lesions 

To evaluate serum levels of creatine phosphokinase (CPK) and creatine kinase-MB fraction (CK-MB)—biomarkers of cardiac lesions—after CIR, blood samples (3–5 mL) were collected from the abdominal aorta and placed in siliconized tubes, exclusively from rats that survived the full 75 min CIR protocol. Blood samples were centrifugated at 2500 rpm, 5 °C for 40 min. The supernatant was separated and stored at –20 °C for subsequent enzymatic analysis. The enzymatic activities of CPK and CK-MB in serum were determined with a kinetic UV test, measured at 340 nm using a commercial kit (VIDA Biotecnologia, Belo Horizonte, MG, Brazil) [22].

### 2.5. Pharmacological Treatments

To evaluate the effects of with ENOX (1 mg/kg, IV; Sanofi Winthrop Industry, France) on the incidence of VA, AVB, and LET caused by CIR, rats were treated with this LMWH alone or combined with drugs that block ABC transporter-mediated cAMP efflux from cardiac cells (probenecid, PROB; Sigma-Aldrich, St. Louis, MO, USA) or an A1R antagonist (8-cyclopentyl-1,3-dipropylxanthine, DPCPX; Sigma-Aldrich, St. Louis, MO, USA) [27,28]. All drugs were intravenously (IV) administered before CIR. We previously showed that LET in control animals treated with 0.9% saline solution (SS) varied from 70 to 80% [23,24]. In this work, 94 animals were used, divided into seven experimental groups:

(1) SHAM-operated: Rats were submitted to the previously described procedure; however, the nylon thread was only passed under the left coronary artery and the coronary artery was not blockaded; therefore, neither ischemia nor reperfusion was induced (n = 10);
(2) CIR + SS: control rats that received the 0.9% SS vehicle (IV) immediately before cardiac ischemia (n = 24);
(3) CIR + ENOX: rats treated with ENOX (1 mg/kg, IV) immediately before cardiac ischemia (n = 12);
(4) CIR + PROB: rats treated with PROB (100 mg/kg, IV) five minutes before cardiac ischemia (n = 12);
(5) CIR + DPCPX: rats treated with DPCPX (100 µg/kg, IV) five minutes before cardiac ischemia (n = 12);
(6) CIR + ENOX + PROB: rats treated with PROB (100 mg/kg, IV) five minutes before cardiac ischemia, and treated with ENOX (1 mg/kg, IV) immediately before cardiac ischemia (n = 12);
(7) CIR + ENOX + DPCPX: rats treated with DPCPX (100 µg/kg, IV) five minutes before cardiac ischemia, and treated with ENOX (1 mg/kg, IV) immediately before cardiac ischemia (n = 12).

### 2.6. Statistical Analysis 

Data corresponding to VA, AVB, and LET incidences were expressed as percentages and statistically analyzed using Fisher’s exact test with the Prism 5.0 software (GraphPad, USA). Data corresponding to creatine phosphokinase (CPK) and MB-fraction creatine kinase (CK-MB) serum levels (U/L) were expressed as mean ± standard error of mean (SEM) and statistically analyzed by an analysis of variance test using Prism. Results were considered statistically significant when *p* < 0.05.

## 3. Results

**Figure 2:**
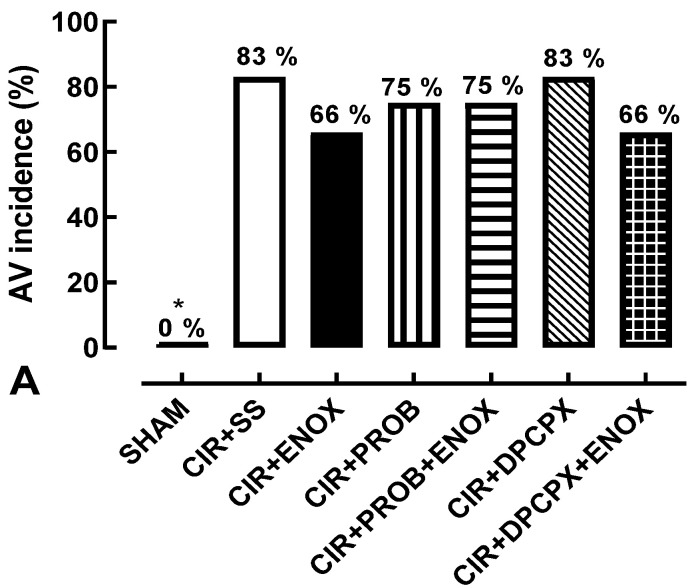

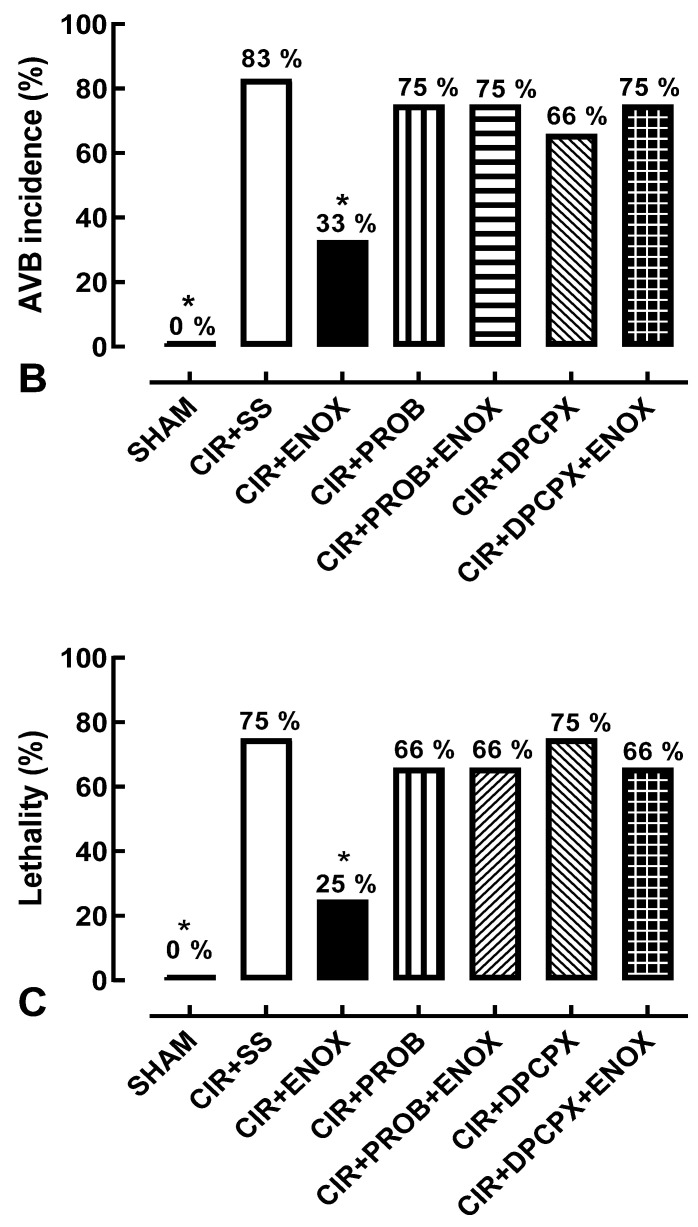
Incidence of (**A**) ventricular arrhythmias (VA); (**B**) atrioventricular block (AVB) and (**C**) lethality (LET) in the SHAM, CIR + SS, CIR + ENOX, CIR + PROB, CIR + PROB + ENOX, and CIR + DPCPX + ENOX groups. The results are expressed as percentage frequencies and compared with Fisher’s exact test (* *p* < 0.05). VA, AVB, and LET were not observed in the SHAM group. ENOX did not reduce the incidence of VA when compared with the control CIR + SS group but did significantly reduce AVB and LET incidence. Disruption of the extracellular cAMP-ADO pathway with either PROB or DPCPX abolished the cardioprotective effect of ENOX on AVB and LET. Treatment with either PROB or DPCPX alone had no effect on mortality. CIR + SS = cardiac ischemia and reperfusion group treated with saline solution; CIR + ENOX = cardiac ischemia and reperfusion group treated with enoxaparin; CIR + PROB = cardiac ischemia and reperfusion group treated with probenecid 5 min before CIR; CIR + PROB + ENOX = cardiac ischemia and reperfusion group treated with probenecid 5 min before treatment with enoxaparin, administered immediately before CIR; CIR + DPCPX + ENOX = cardiac ischemia and reperfusion group treated with DPCPX 5 min before treatment with enoxaparin, administered immediately before CIR.

The animals in the SHAM group did not show VA, AVB, and LET; in addition, the serum levels of biochemical markers of cardiac injury in the SHAM group were lower when compared with the other groups, results that prove that the CIR protocol was able to promote both electrical alterations that were observed in the different forms of cardiac arrhythmias and cardiac injuries proven by serum increases in the markers of myocardial injury—CPK and CK-MB. The animals in the SHAM group did not have VA, AVB, and LET, that is, VA (0%), AVB (0%), and LET (0%) (Figure 2).

In all experimental groups studied, cardiac rhythm before CIR was registered and it was used to validate this animal CIR model to study the pharmacological effects produced by treatment with ENOX (1 mg/kg, IV, for one minute before CIR) on the incidence of VA, AVB, and LET induced by CIR. 

Figure 2A shows that VA incidence in the CIR + ENOX group (66%) was not statistically different from the CIR + SS (83%), CIR + PROB (75%), CIR + ENOX + PROB (75%), CIR + DPCPX (83%), or CIR + ENOX + DPCPX (66%) groups, indicating that these treatments did not interfere with the incidence of AV. However, treatment with ENOX significantly reduced the incidence of AVB and LET induced by CIR. Figure 2B shows that AVB incidence was statistically lower in the ENOX group (33%) compared with the CIR + SS group (83%). Figure 2C shows that LET incidence was also statistically lower in the CIR + ENOX (25%) group compared with the CIR + SS group (75%). These results suggest that treatment with ENOX reduced the incidence of severe and fatal arrhythmias caused by CIR, confirming the cardioprotective activity of this LMWH. 

Pretreatment with drugs DPCPX and PROB abolished the effects produced by treatment with ENOX. Figure 2B shows that AVB incidence was similar in the CIR + ENOX + PROB (75%) and CIR + ENOX + DPCPX (75%) groups compared with the CIR + PROB (75%) and CIR + DPCPX (66%) groups, respectively. Figure 2C shows that LET incidence was also similar in the CIR + ENOX + PROB (66%) and CIR + ENOX + DPCPX (66%) groups compared with the CIR + PROB (66%) and CIR + DPCPX (75%) groups, respectively. These results suggest that treatment with PROB and DPCPX inhibited the cardioprotective effects produced by ENOX treatment, suggesting that pharmacological modulation of the purinergic pathway by ENOX in cardiac cells is involved in its cardioprotective effects.

**Figure 3:**
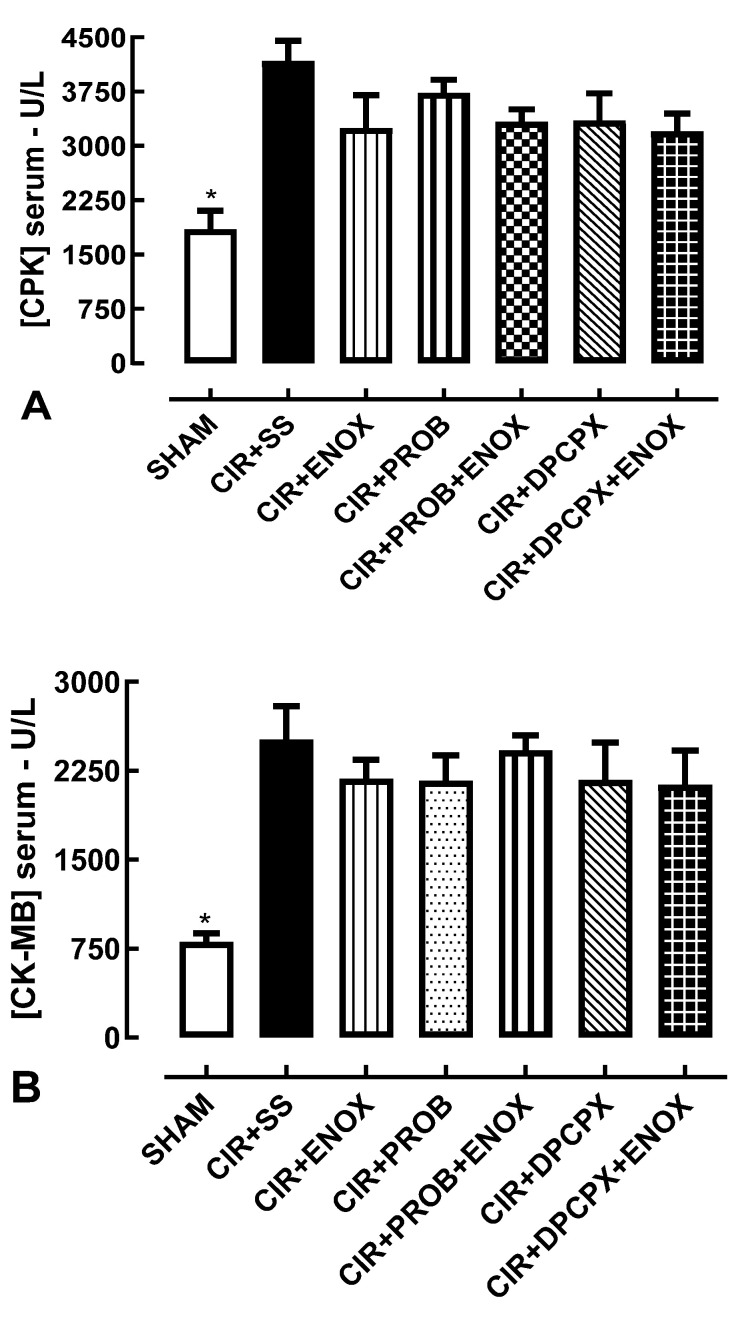
Serum concentration of CPK (**A**), and CK-MB (**B**) in the CIR + SS, CIR + ENOX, CIR + PROB, CIR + PROB + ENOX, and CIR + DPCPX + ENOX groups. The results are expressed as mean ± standard deviation of the mean, and analysis of variance (ANOVA) was applied, followed by the Tukey post-test (* *p* < 0.05). Serum levels of biochemical markers of cardiac injury in the SHAM group were lower than in the other groups, both CPK and CK-MB. CIR + SS = cardiac ischemia and reperfusion group treated with saline solution; CIR + ENOX = cardiac ischemia and reperfusion group treated with enoxaparin; CIR + PROB = cardiac ischemia and reperfusion group treated with probenecid 5 min before induction of CIR; CIR + PROB + ENOX = cardiac ischemia and reperfusion group treated with probenecid 5 min before treatment with enoxaparin, administered immediately before induction of CIR; CIR + DPCPX + ENOX = cardiac ischemia and reperfusion group treated with DPCPX 5 min before treatment with enoxaparin, administered immediately before induction of CIR.

Figure 3A shows that serum levels of CPK in the SHAM (1846 ± 258.6 U/L) group were statistically different from the other groups, but the CIR + ENOX (3244 ± 456.7 U/L) group did not change significantly when compared with the CIR + SS (4169 ± 567.4 U/L), CIR + PROB (3732 ± 439 U/L), CIR + PROB + ENOX (3330 ± 419 U/L), CIR + DPCPX (3346 ± 761 U/L), and CIR + DPCPX + ENOX (3196 ± 503 U/L) groups. Figure 3B shows that serum levels of CK-MB in the SHAM (807.8 ± 40.3 U/L) group were statistically different from the other groups, but the CIR + ENOX (2370 ± 285.2 U/L) group was also similar compared with the CIR + SS (2511 ± 283.3 U/L), CIR + PROB (2166 ± 213.6 U/L), CIR + PROB + ENOX (2422 ± 126.8 U/L), CIR + DPCPX (2174 ± 314.1 U/L), and CIR + DPCPX + ENOX (2132 ± 289.7 U/L) groups. These results indicated that the cardioprotective effects produced by treatment with ENOX were not directly related to the reduction in cardiac lesion markers, specifically CPK and CK-MB. 

## 4. Discussion

Clinical studies have shown that treatment with LMWHs significantly reduce mortality and reinfarction in patients with AMI [29]. However, the cellular and molecular mechanisms involved in these cardioprotective responses need to be better explored. To advance our knowledge of these mechanisms, we investigated whether treatment with ENOX could be effective and safe to reduce the incidence of severe and fatal arrhythmias induced by CIR. In addition, we investigated whether the pharmacological modulation of purinergic signaling in cardiac cells could be involved in the cardioprotective effects of ENOX. Using an animal CIR model, we showed that treatment with ENOX (1 mg/kg, IV) before CIR significantly reduced the incidence of AVB (from 83% to 33%) and LET (from 75% to 25%) caused by CIR (see Figure 2). Our results indicate that treatment with ENOX before CIR prevents severe and lethal arrhythmias caused by CIR. Studies using the combined treatment of ENOX with the drugs DPCPX and PROB suggest that this LMWH can pharmacologically modulate ADO signaling in cardiac cells, reducing the severe and lethal arrhythmias caused by CIR. These results support the idea that treatment with ENOX could be an effective and safe cardioprotective strategy in AMI therapy in humans. 

Post-AMI patients show increased serum levels of biochemical markers of cardiac injury. CPK and CK-MB have been associated with poor patient prognosis and severe and often fatal ventricular arrhythmias [30]. These myocardial injuries also compromise cardiac output and may lead to atrial arrhythmias, as well as cardiogenic shock [30]. Although the anticoagulant effect of heparin derivatives is crucial for their therapeutic role in these patients, as previously mentioned, it is likely that their cardioprotective effects are also important to prevent sudden death due to cardiac arrhythmias [31]. However, the results obtained in this work showed that treatment with ENOX did not alter the serum levels of the biochemical markers CPK and CK-MB of cardiac injury.

The idea that cardioprotection stimulated by LMWHs could be effective and safe in human AMI therapy is supported by numerous clinical and experimental studies [17,18,19,30,31,32,33]. In addition to anticoagulant activity, it has been proposed that LMWHs may act on multiple cell mechanisms involved in the regulation of cardiac chronotropism and inotropism, attenuating ionic imbalance and mitochondrial bioenergetic collapse, and thus reducing the incidence of severe and fatal arrhythmias [17].

Other pharmacological properties of LMWHs, such as antithrombotic and anti-inflammatory and activities, could also contribute to their cardioprotective effects, acting as “multi-target” drugs [17,18,31,32,33,34]. Inflammation and inflammatory mediators, in particular, have been implicated in CIR-induced injuries and arrhythmias [35]. In cardiopulmonary bypass patients, heparin has already been successfully used to increase the biocompatibility of the device and reduce complement factor C3b/c and elastase-alpha [1]–antitrypsin complex levels [35].

In vivo studies have shown that treatment with oligo-HLMWH (2 kDa), administered 10 or 30 min before CIR (ischemia for 5 min and reperfusion for 5 min) at doses of 5 to 30 mg/kg (IV), significantly reduced VA incidence and LET caused by CIR in rats [19]. It was also shown that treatment with 2-O, 3-O desulphated heparin (ODSH) (15 mg/kg, IV), administered 5 min before reperfusion for 180 min preceded by ischemia for 75 min, significantly reduced myocardial damage caused by CIR in pigs [32]. In this study, while ENOX protected rats subjected to CIR from AVB or death, we did not see an effect on VA. Previously, our own group observed a protective effect of LMWHs on ventricular arrhythmias [18]. However, this effect was seen in isolated hearts, and may not directly translate in vivo. While Guarini et al. (1995) observed significant reduction in ventricular arrhythmias with a LMWH in vivo, their experimental protocol was 5-min ischemia/5-min reperfusion, while our study evaluated the effects of ENOX on more prolonged ischemia (10 min) and, more importantly, considerably longer reperfusion (75 min). It is possible that longer periods of ischemia-reperfusion lead to more severe arrhythmias and injuries, thus attenuating some of the cardioprotective effects of LMWHs. In addition, the different doses used—20 mg/kg oligo-H and 1 mg/kg ENOX—make direct comparison of these observations particularly difficult.

Our previously published in vitro work has shown that LMWHs, such as ENOX, can reverse electrically induced arrhythmias in the right atrium and in isolated rat heart, reinforcing their cardioprotective effect [18]. LMWHs reduce the excitability of the myocardium due to their actions on the ionic transport mediated by plasmalemmal Ca^2+^ and Na^+^ channels in cardiac cells [36,37,38]. It is well established that Ca^2+^ transport mediated by the plasmalemmal Na^+^-Ca^2+^ exchanger (NCX) plays an important role in the regulation of the cardiac coupling of excitation–contraction due to its crucial role in the fine regulation of c[Ca^2+^] [39]. Physiological NCX activity is responsible for removing up to 20% of the Ca^2+^ present in the cytosol immediately after contraction in mammalian cardiac cells [39], but altered NCX activity in CIR contributes to the genesis of cardiac arrhythmias [37,38,40,41,42]. Thus, pharmacological inhibition of the plasmalemmal NCX (reverse mode) by LMWHs can reduce the cardiac damage caused by CIR [18,42].

Cardiac hypoxia caused by CIR results in, among other cellular alterations, a severe deficit in mitochondrial ATP production, which drastically reduces plasmalemmal Na^+^/K^+^ ATPase activity. This action results in increased cytosolic Na^+^ (c[Na^+^]) and consequent reversion of the direction of Na^+^ and Ca^2+^ transport by the plasmalemmal NCX, generating cytosolic Ca^2+^ overload due to the increased influx of this ion by the operation of the reverse mode NCX [3,43]. The persistence of this ionic imbalance through a prolonged period favors the increased incidence of severe and fatal arrhythmias during ischemia and the onset of reperfusion channels [3,38,43,44]. For instance, Ca^2+^ overload and ATP depletion increase the activity of the TRPM4 cation channel, which has been implicated in fatal CIR-induced arrhythmias [45]. In this regard, several studies have proposed that pharmacological modulation of plasmalemmal NCX could attenuate the ionic imbalance in cardiac cells, reducing the incidence of severe arrhythmias caused by CIR [17,18,22,40].

In addition to this action on the plasmalemmal NCX, LMWHs can stimulate cAMP transport from cardiac cells to the extracellular medium, where they are rapidly converted by ecto-phosphodiesterases and ectonucleotides to ADO that, in turn, activates cardiac A1R resulting in the attenuation of the positive chronotropic response produced by stimulation of cardiac β1-adrenoceptors (β1-AR) by catecholamines or other stimuli that increase cardiac activity [25,46,47,48]. It has been proposed that this modulatory role of A1R on cardiac function finely adjusts cardiac chronotropism, and thus reduces the incidence of cardiac arrhythmias [5,17,49]. It has also been suggested that this cardioprotective action of ADO mediated by cardiac A1R could occur in different physiological and pathological conditions, including CIR and cardiac pre- and post-ischemic conditioning [17,18,50,51]. Interestingly, heparin and LMWHs can prevent cardiac arrhythmias associated with sudden death caused by ventricular tachycardia, *torsades de pointes*, and ventricular fibrillation, and first- and second-degree AVB in animal CIR models, as well as in isolated rat atria [17,18,52]. Since Ca^2+^ acts as a second intracellular messenger responsible for the regulation of various enzymes in cardiac cells, including AC, intracellular Ca^2+^ overload may also indirectly promote myocardial injuries and lethal arrhythmias by disruption of the cardioprotective cAMP–ADO pathway [17].

The activation of cardiac β1-AR considerably increases intracellular cAMP levels in cardiac cells when extracellular Ca^2+^ is decreased [25,47,53] or when the plasmalemmal NCX is inhibited in these cells [40,52]. cAMP, in turn, regulates c[Ca^2+^] in cardiac cells and its dysfunction plays an important pathological role in the cardiac inflammation induced by AMI [27,54]. In addition, cAMP is involved in other regulatory mechanisms in cardiac cells. For example, Sellers et al. [48] showed that cAMP efflux mediated by multidrug resistance proteins transporters (MRPTs) in cardiac cells promotes the increment of extracellular ADO concentration resulting in an important attenuation of the β1-AR activity in these cells, and consequently a reduction in the contractile activity of the myocardium.

**Figure 4:**
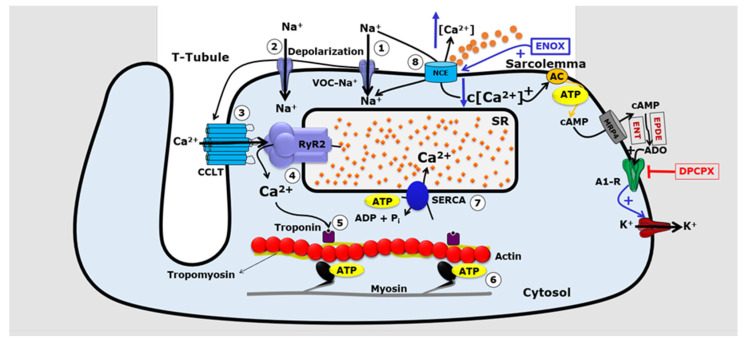
Molecular mechanisms involved in enoxaparin (ENOX) regulation of cardiac cell function. ENOX acts on the intracellular production and efflux of second-messenger cAMP, which is later transformed by extracellular enzymes into adenosine (ADO), which in turn is an antiarrhythmic through the activation of A1R receptors. ENOX stimulates the Na^+^-Ca^2+^ exchanger (NCX) and, therefore, reduces cytosolic calcium in cardiac cells. In addition, ENOX is also able to block voltage-gated calcium channels, which also causes a decrease in cytosolic calcium. This reduction in cytosolic calcium in cardiomyocytes increases intracellular levels of cAMP through the activation of adenylate cyclase (AC), a membrane enzyme. cAMP activates PKA, which phosphorylates L-type Cav (L-Cav), resulting in increased Ca^2+^ influx with positive chronotropic and inotropic responses. The increase in intracellular cAMP stimulates the transport of cAMP to the extracellular medium through the MRP4 ABC transporter. In the extracellular environment, cAMP is converted to ADO due to the enzymatic action of ectophosphodiesterases (EPDE) and ectonucleotidaeses (ENT). This purinergic signaling involves the action of ADO on A1R which modulates voltage-gated potassium channels in cardiac cells.

Menezes-Rodrigues et al. [25] and Sassi et al. [47] showed that the blockade of β1-AR in cardiac cells significantly attenuated the negative inotropic effect of extracellular ADO resulting from MRPT-mediated cAMP efflux from muscle cells, thus highlighting the importance of this regulatory mechanism of cardiac function [27,52]. Here, we propose that the antiarrhythmic effects produced by heparins and LMWHs are due to the pharmacological modulation of ADO signaling in cardiac cells. Figure 4 shows a schematic representation of pharmacological modulation of this signaling.

We suggest that this strategy could be useful to prevent sudden death due to severe arrhythmias caused by cardiac collapse in patients with AMI. It is important to note that pharmacological activation of cardiac A1R reduces the excitability of cardiac cells [28,50], possibly reducing the probability of lethal atrioventricular blocks. In this study we demonstrate that, at least in part, the antiarrhythmic effect of ENOX is due to its action on the cAMP/ADO extracellular pathway in cardiac cells, preventing arrhythmias and death caused by cardiac collapse in patients with AMI.

The increase in extracellular levels of ADO generated by the enzymatic degradation of ATP released from intracardiac sympathetic neurons, combined with the transport of cAMP to the extracellular medium from cardiac cells during stimulation, produces an increase in the activation of cardiac A1R and attenuates the positive chronotropic response stimulated by β1AR [17,50]. Several lines of evidence suggest that this adrenergic–purinergic communication involved in the regulation of cardiac chronotropism contributes importantly to cardioprotective responses in different pathophysiological conditions [17,46,47]. In the present work, we showed that the A1R-antagonist DPCPX blocked the cardioprotective effects of ENOX, indicating the involvement of this ADO receptor in these effects.

## 5. Conclusions

The results obtained in this work indicate that treatment with ENOX was effective to prevent severe and lethal arrhythmias induced by CIR due to the pharmacological modulation of ADO signaling in cardiac cells, suggesting that this cardioprotective strategy could be promising in AMI therapy.

## Data Availability

The data will be available upon justified request and agreement of the authors.
